# Expression of Odorant Receptor Family, Type 2 OR in the Aquatic Olfactory Cavity of Amphibian Frog *Xenopus tropicalis*

**DOI:** 10.1371/journal.pone.0033922

**Published:** 2012-04-11

**Authors:** Tosikazu Amano, Jean Gascuel

**Affiliations:** 1 CNRS, UMR6265 Centre des Sciences du Goût et de l'Alimentation, Dijon, France; 2 INRA, UMR1324 Centre des Sciences du Goût et de l'Alimentation, Dijon, France; 3 Université de Bourgogne, UMR Centre des Sciences du Goût et de l'Alimentation, Dijon, France; Duke University, United States of America

## Abstract

Recent genome wide *in silico* analyses discovered a new family (type 2 or family H) of odorant receptors (ORs) in teleost fish and frogs. However, since there is no evidence of the expression of these novel OR genes in olfactory sensory neurons (OSN), it remains unknown if type 2 ORs (OR2) function as odorant receptors. In this study, we examined expression of OR2 genes in the frog *Xenopus tropicalis*. The overall gene expression pattern is highly complex and differs depending on the gene and developmental stage. RT-PCR analysis in larvae showed that all of the OR2η genes we identified were expressed in the peripheral olfactory system and some were detected in the brain and skin. Whole mount *in situ* hybridization of the larval olfactory cavity confirmed that at least two OR2η genes so far tested are expressed in the OSN. Because tadpoles are aquatic animals, OR2η genes are probably involved in aquatic olfaction. In adults, OR2η genes are expressed in the nose, brain, and testes to different degrees depending on the genes. OR2η expression in the olfactory system is restricted to the medium cavity, which participates in the detection of water-soluble odorants, suggesting that OR2ηs function as receptors for water-soluble odorants. Moreover, the fact that several OR2ηs are significantly expressed in non-olfactory organs suggests unknown roles in a range of biological processes other than putative odorant receptor functions.

## Introduction

Olfaction is essential for animal survival to find food and mating partners, and to escape from predators. To recognize the huge variety of odorant molecules in the environment, there are large numbers of odorant receptors (ORs) which often make up the largest gene family in the tetrapod genome [1]. For example, the human and mouse genome contains >800 [2], [3] and ∼1400 [4], [5] OR genes, respectively, including nonfunctional genes. There are 388 intact OR genes in humans [6], [7] and 1037 in mice [6], and >800 OR genes are expressed in mouse olfactory epithelium (OE) [8]. In the amphibian frog *Xenopus tropicalis*, more than 1500 OR genes have been identified in the genome [9].

ORs have been classified into two groups [10]. Class I is occasionally referred to as fish-like since this group was initially found in teleost fish. These are thought to function as receptors for water-soluble odorants [10]–[13]. Tetrapod-specific class II receptors may play a role in the detection of air-borne odorants [10]–[12]. Phylogenetic analyses showed that class I and II ORs made up one large gene family (type 1, (OR1)) that could be divided into several subgroups α, β, δ, ε, ζ (class I), and γ (class II) [14]. Bioinformatic studies also revealed that the mammalian genome contained a number of class I ORs [5]. These ORs mainly belong to the α subgroup, which is not found in the fish genome [14]. Thus, class I ORαs are thought to recognize air-borne odorants [15]. Recent genome-wide screening of G-protein coupled receptor genes discovered another type of ORs named type 2 (OR2) in the fish and the frog [14] or family H in the fish, which corresponds to OR2η, one of three subgroups of OR2 [16]. OR2s are thought to act as receptors for odorants, even though the function of the OR2 is not clear since no evidence of their expression in olfactory sensory neurons (OSNs) is available. Only one gene OR137-7 (a member of the family H) is known to be expressed in the olfactory epithelium (OE) in zebrafish [9], [16].

*Xenopus* adapts to both aquatic and terrestrial life. During the early larval period before metamorphosis, there is a pair of single olfactory cavities (OCs) which specifically recognize water-soluble odorants [17], [18]. The adult frog has a pair of two distinct olfactory cavities, an air-filled cavity (principal cavity, PC) and a water-filled cavity (medium cavity, MC), which are separated by a valve [17], [19]. The surface of the OE in the PC is covered by mucus containing olfactory binding protein (OBP) [20], which is similar to mammalian OE [21], [22]. Although its exact functions are unclear, OBP is thought to be an adaptation of olfaction to odorant detection in the air [23], [24]. Thus, it is thought that the PC and the MC participate in the recognition of air-borne odorants and water-soluble odorants, respectively. This unique feature of the *Xenopus* olfactory system gives the opportunity to study OR functions. To clarify the chemosensory function of OR2 genes, it is necessary to localize OR2 gene expression in the OSN. Thus, our study aimed to reveal OR2 expression in the frog.

In this paper, we showed that the overall pattern of OR2 gene expression was highly complex and differed according to the gene and the developmental stage. All of the OR2η genes we examined were expressed in the olfactory organ both in the larva and the adult with different expression levels. Moreover, at least two of the OR2η genes so far tested were expressed in the OSNs in the larval OC. Altogether, this is the first evidence of OR2 expression in the OSNs, which support the idea of the putative olfactory function deduced from their predicted protein sequence [16]. In the adult nose, OR2ηs were preferentially expressed in the MC. In addition, because some OR2ηs were also expressed in the brain and skin in the larva, and the brain and testes in the adult, involvement of OR2η in non-olfaction processes also has to be considered.

## Materials and Methods

### Bioinformatics

XtOR2 genes were collected from the latest version of the *X. tropicalis* genome draft (JGI, version 4.1, http://genome.jgi-psf.org/Xentr4/Xentr4.home.html), by BLAST using published *X. tropicalis* OR2 gene sequences [14] in the previous version of the genome draft (JGI, version 3.1) as a query. Multiple nucleotide sequence alignments were performed using a web-base program (MAFFT version 6, http://mafft.cbrc.jp/alignment/server/index.html) using default parameters. The phylogenetic tree was constructed using the neighbor-joining method [25]. Three *X. tropicalis* melanocortin receptors were used as an out group. The reliability of each tree node was tested by the bootstrap method with 1000 replications. The amino acid sequence homology analysis was done using MAFFT.

### Animals, RNA extraction and PCR

All experimental procedures were submitted to both the French veterinary committee (DSV: Direction des services vétérinaires), and to the local ethics committee of Burgundy University. The experimental procedures were approved by these committees (approval numbers are respectively: DSV accreditation: 21CAE 016, and ethics committee: G04bis, H04bis, I04bis).

*X. tropicalis* tadpoles were staged according to Nieuwkoop and Faber [26]. The olfactory cavities and other organs were dissected from the staged tadpoles and the sexually mature adult frogs. The PC and the MC were separated surgically from the adult frogs. Contamination of the PC tissue in the MC preparation was checked by the detection of OBP RNA, which is specifically expressed at an extremely high level in the PC (see results section).

Total RNAs were extracted from the organs using TRIzol (Invitrogen) and cleaned using the RNeasy kit (Qiagen). DNase I-digested RNA (0.5 or 1 µg) was used for cDNA synthesis (Invitrogen). cDNA from 0.4 ng RNA was used for a 10 µl PCR reaction. No genomic DNA contamination was confirmed by no reverse transcriptase control in the cDNA synthesis reactions. To confirm equal amounts of cDNA for the PCR reaction we used a ubiquitously and constantly expressed ribosomal protein gene, rpL8 for endogenous control [27]. Quantitative real time PCR (qPCR) was done using the SYBR green method (Takara). The Amplification efficiency of each gene specific primer set was tested by using the genomic DNA. Only primer sets which amplified the genomic DNA with similar efficiency were used for further experiments. Because of extremely the high homology between XtOR2η2 and 2η3, and XtOR2η7 and 2η8, respectively, we used the primer set which amplified both XtOR2η2 and 2η3, and both XtOR2η7 and 2η8, respectively. We confirmed by sequencing that all primer sets we used amplified the given OR2 gene species. To normalize OR expression in the OC in each sample we used olfactory marker protein (OMP) as an internal control since OMP is known to be ubiquitously expressed in mature OSN [28]. Although in *X. laevis* there are two olfactory marker proteins, which show distinct expression pattern in the OC [29], we only found one OMP gene (GeneBank accession no NM_203734) in the *X. tropicalis* genome. The primer sequences we used were: XtOR2κ1 (5′-TGTATCTACCTGGTGGACTTCTTG-3′, 5′-AATAAAGTCAGGTACGTTAGGTGG-3′), XtOR2θ1 (5′-GTTCACCTTTCAACAAAACCTCAG-3′, 5′-TTCTTTAGTATGGCCACTAGAACC-3′), XtOR2η1 (5′-TGTTGAACACCACGTCGCTCTACG-3′, 5′-GAACACCTTCAGGATCACCGACAC-3′). XtOR2η2/3 (5′-CTCATATGCTCGTTAGTGACACCC-3′, 5′-GTAACGCGGTAAGTGACCGTAGCC-3′), XtOR2η4 (5′-CTACTGTCATTGTGTTGCTGCTGC-3′, 5′-GTGGATGAATAGGACGTATCGGAC-3′), XtOR2η5 (5′-CAGCAAGACCGGAGAGATCGTGAG-3′, 5′-TGGGCAAAGAGTACATAGCGGGAC-3′), XtOR2η6 (5′-TTTTCTTCACCAAACCTTCTCTGC-3′, 5′-GTGATTATAGCCAAGTAAAGCGTC-3′), XtOR2η7/8 (5′-ATCGTGATCCTCACCCTCGTTTGC-3′, 5′-AATGGCACGGGGAGGTATATCAGG-3′), class I ORα5 (5′-CAAAATGACTCGGCTCTTCAGGAG-3′, 5′-AACAAGACCAGTATTTCACTGCTG-3′), δ15 (5′-ACTCAATTTTTCCATTTTCATGGC-3′, 5′-CCCATTATAGACAGTATTGTGAAG-3′), ε10 (5′-TGGCATATTCCCTACTTTCTACTG-3′, 5′-CCAATAAGCTACTGACCCAGTCTG-3′), class II ORs (5′-TCCTTGGAACCCTGGCATGTCTGG-3′, 5′-GTAGATCTGAGTTATACAAGCTGG-3′)(5′-TCCATTATGGGTTTCAGGCTTTGC-3′, 5′-TGATCTATAACAAATGGGCCACAG-3′ v2), rpL8 (5′-GGCTCTGTTTTTAAAGCCCACGTC-3′, 5′-CAGGATGGGTTTGTCAATACGACC-3′), OMP (5′-TCTATCGGCTGGATTTCTCCAAGC-3′, 5′-AACATTTGATGGCGGACGGGTCGG-3′), OBP (5′-ATGAAGGCGGAGATGAAGACGGAG-3′, 5′-TGTCTTCCTTCAGGCCCAGCTTTA-3′).

### Whole mount in situ hybridization

Whole mount *in situ* hybridization (WISH) on stage 47 larvae was carried out as described previously [30], and digoxigenin labeled and fluorescein labeled probes were detected by using the TSA plus fluorescence system (PerkinElmer). A cRNA probe was synthesized by T7 RNA polymerase (Promega) from a PCR fragment containing 3′-UTR of XtOR2η4 and XtOR2η5 ligated to a T7 promoter. Primers used for amplification of XtOR2η4 and XtOR2η5 3′-UTR were 5′-TACTGTATGTGTGTGTGATAGTCC-3′ and 5′-TTTGGCCAAATACCTACTGCTGAG-3′, 5′- ATTACGGATTCCGTCAGCTTCAC-3′ and 5′- ATTTGTATGGGTTGCAGTTGCTG-3′, respectively.

## Results

### OR2 genes in the X. tropicalis genome

**Figure 1:**
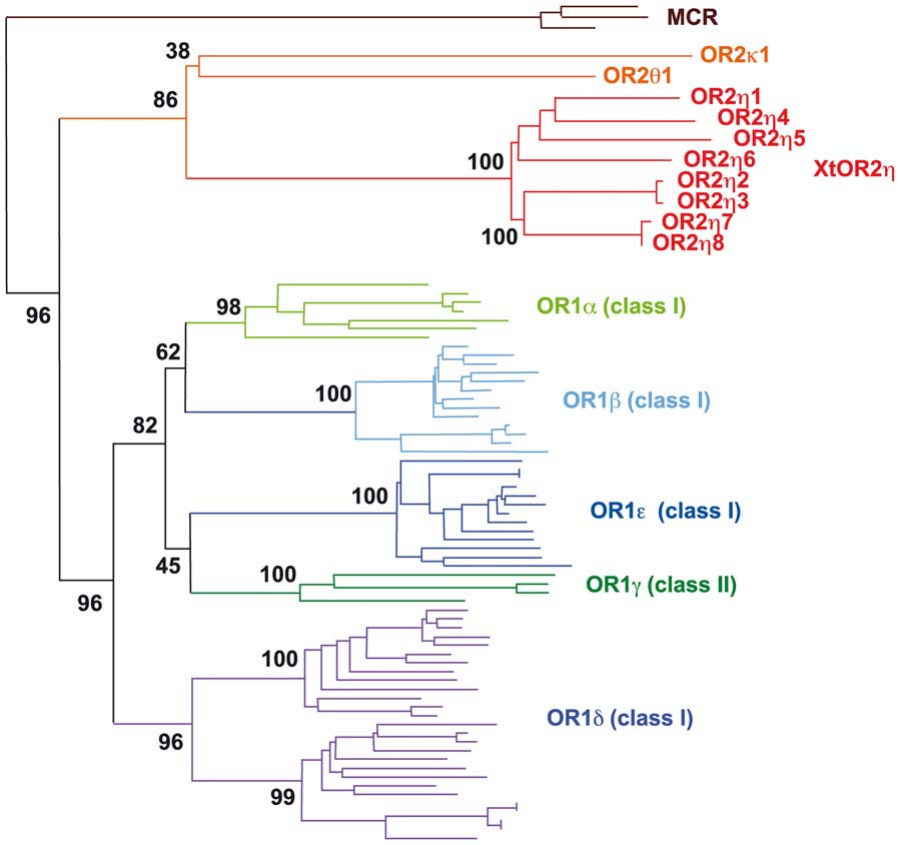
Phylogenetic tree of *X. tropicalis* OR1 and OR2 genes. Amino acid sequences of all XtOR2 and all XtOR1 class I (OR(I)), four XtOR1 class II ORs (OR(II)γ), and three melanocortin receptors (MCRs), were used for the phylogenetic analysis.

We identified 10 intact XtOR2 genes in the genome by BLAST using published sequences of *X. tropicalis* OR2 (XtOR2) genes [14] as a query, (Table 1). They were clearly separated from the classical OR1 genes (class I and II), and divided into 3 groups (Table 2, Fig. 1) as previously reported [9], [14]. We described these genes according to the nomenclature proposed by Glusman *et al*. [31] with minor modifications to adapt to the most recent classification of OR genes in the frog and the fish as follows: XtOR2η1, *X. tropicalis* | Odorant Receptor | type 2 | group η | individual gene number 1 in the group. Both group κ and θ contained a single copy gene and were located on scaffold 55 (JGI ver.4.1), but were separated by many non-OR genes. The largest group, η, consisted of 8 genes. Seven genes of group η (OR2η1-7) were mapped on a single scaffold, 982, making a gene cluster (Table 1) and one (OR2η8) was on another scaffold, 1014. Both were surrounded by different sets of non-OR genes indicating that XtOR2η8 was located outside the XtOR2η cluster in the genome. Very recently, basically similar results were obtained by Niimura [9]. This study identified 14 OR2 genes (1κ, 4θ, 12η including two pseudogenes and one truncated gene) in the *X. tropicalis* genome. All the OR2 genes we identified were included in this group. This small difference in the number of genes might have been due to a different parameter setting for the BLAST search.

**Table 1:** X. tropicalis OR Type 2 genes.

Gene name	Scaffold	Position	'+ or −*	size**	Gene ID	Protein ID	Another name***
XtOR2κ1	55	340794–341681	−	295	e_gw1.55.204.1	320008	Xetr-κ1
XtOR2θ1	55	2538054–2539091	+	344	gw1.55.343.1 (part)	143443	Xetr-θ1.1
XtOR2η1	982	80133–81065	−	310	fgenesh1_pg.C_scaffold_982000006	188437	ORs982.1
XtOR2η2	982	93665–94591	−	308	none	none	ORs982.3
XtOR2η3	982	117240–118166	−	308	fgenesh1_pg.C_scaffold_982000010	188441	ORs982.5
XtOR2η4	982	124362–125276	−	304	fgenesh1_pg.C_scaffold_982000012	188443	ORs982.6
XtOR2η5	982	134704–135672	−	322	fgenesh1_pg.C_scaffold_982000013	188444	ORs982.7
XtOR2η6	982	150541–153538	−	319	fgenesh1_pg.C_scaffold_982000014 (cDNA)	188445	ORs982.8
XtOR2η7	982	166464–167351	−	295	fgenesh1_pg.C_scaffold_982000015	188446	ORs982.9
XtOR2η8	1014	161856–162743	−	295	fgenesh1_pg.C_scaffold_1014000016	188695	ORs1014.2

* orientation.

** amino acid length.

*** annotated by Niimura (9).

**Table 2:** Amino acid sequence identity (%) of *X. tropicalis* type 2 ORs and class I Ors.

	XtOR2κ1	XtOR2θ1	XtOR2η1	XtOR2η2	XtOR2η3	XtOR2η4	XtOR2η5	XtOR2η6	XtOR2η7	XtOR2η8	MCR	XtOR1(I)α1	XtOR1(I)δ1
XtOR2κ1													
XtOR2θ1	20												
XtOR2η1	15	17											
XtOR2η2	18	16	47										
XtOR2η3	19	16	46	93									
XtOR2η4	15	15	47	46	46								
XtOR2η5	17	16	41	39	39	46							
XtOR2η6	17	18	42	44	44	42	39						
XtOR2η7	19	20	48	50	50	45	41	44					
XtOR2η8	19	20	48	50	50	45	41	44	100				
MCR	13	16	14	14	15	13	13	13	17	17			
XtOR1(I)α1*	12	18	19	19	16	14	16	16	16	16	18		
XtOR1(I)δ1**	16	17	14	14	16	15	15	15	18	18	14	29	

* XtOR1(I)α1: estEXT_fgenesh1.pg.C_5720016.

** XtOR1(I)δ1: e_gw1.799.21.1.

### Overall pattern of expression of XtOR in the tadpole and adult

**Figure 2:**
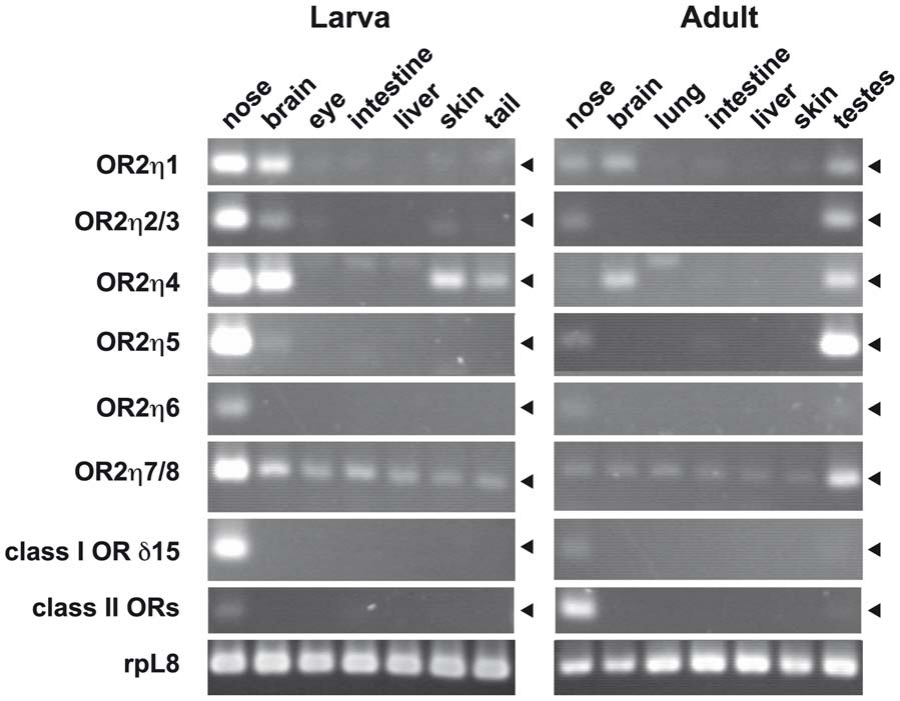
XtOR2 gene expression in various organs in stage 55 larvae and full-grown adults. The primer set for class II OR amplified multiple class II OR genes. PCR cycles were adjusted to obtain adequate amounts of the products (35 cycles for OR2 and class I ORs, and 30 cycles for rpL8 and class II ORs).

To understand the putative function of OR2 receptors, we first examined the expression of all of the XtOR2 genes we identified in various organs in the larval and adult animals by RT-PCR. The expression of XtOR2κ1 and XtOR2θ1 was hardly detected in the olfactory system in both the larval and the adult animals (Data not shown). We therefore focused on XtOR2η expression. The RT-PCR analysis in the organs of XtOR2η genes demonstrated a variety of expression patterns (Fig. 2). In the larva (Fig. 2, left panel), two, out of six, XtOR2η genes were expressed only in the nose, one at a high expression level (OR2η5) and the other at a low level (OR2η6). XtOR2η1, 2/3 and 4 RNAs were detected, not only in the nose, but also at various levels in the brain. OR2η4 was also expressed in the skin and the tail. Since the tail contained the skin, the signal in the tail might be due to the skin of the tail. Besides the strong expression of XtORη7/8 in the nose, these two genes were also expressed at a low level in all of the organs tested.

The expression pattern of these genes in the adult frog, was different from that in the tadpole (Fig. 2, right panel). Expression in the nose and the brain of the adult was much lower than in the larva. Interestingly, all XtOR2η genes except for XtOR2η6 were expressed at various levels in the testes.

Like for the class I OR that we examined here, the expression of XtOR2η in the nose was stronger in the larva than in the adult whereas class II OR expression was strongly up-regulated in the adult nose.

### Respective expression between the PC and MC in adult

**Figure 3:**
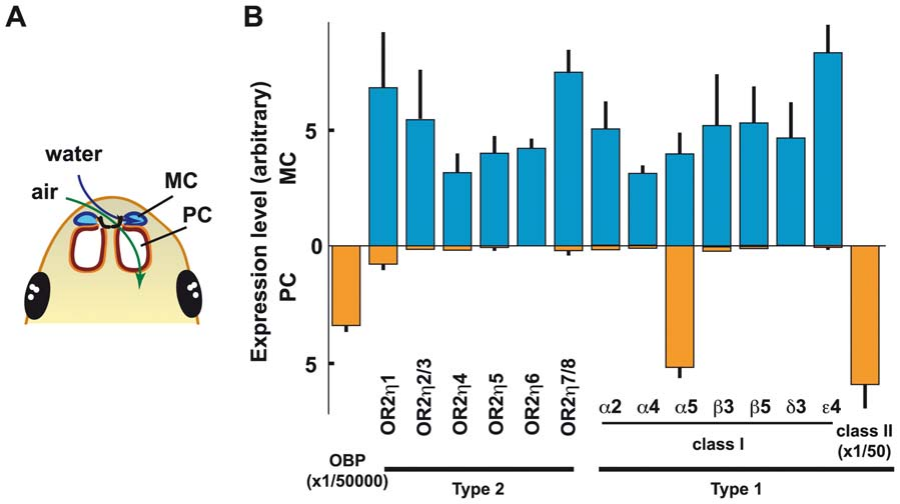
OR gene expression in adult olfactory cavities. A: Schematic illustration of the nasal cavities of the adult frog. The MC is filled in water and the PC is open to air. The air flow goes though the PC to the lung. B: The expression of OR2ηs and some OR1s (class I and II) in adult nasal cavities. Quantitative PCR was done for each OR gene and normalized by using the OMP gene, which is expressed in every mature OSN. Most class I ORs were preferentially expressed in the MC. Class I ORα5 was exceptionally expressed at a significant level in the PC as well as the MC. Bars represent standard deviation (n = 3). Note that the expression level of OBP and class II (mix) OR was much higher than each single OR2η and class I OR gene (different scale). Gene ID: OR1α2: ENSXETG00000024801.1, OR1α4: fgenesh1_pg.C_scaffold_1078000009, OR1α5: e_gw1.2098.6.1, OR1β3: fgenesh1_pg.C_scaffold_976000003, OR1β5: e_gw1.976.19.1, OR1δ2: e_gw1.799.9.1, OR1ε4: e_gw1.799.69.1.

The adult frog has two distinct OCs, the PC and the MC (Fig. 3A), which are involved in the detection of air-borne and water-soluble odorants, respectively [19]. It has been shown that the surface of the sensory epithelium in the PC of the adult frog is covered with OBP which could be considered a marker for the aerial olfactory system [20]. Our results confirmed that the OBP gene was exclusively detected in the PC [20] at an extremely high level (approximately 15,000 times higher than the OMP, Fig. 3B). OBP RNA was hardly detected in the MC (Fig. 3B), indicating that the MC preparation did not contain a significant amount of PC tissue contamination. In contrast, most class I OR genes (we examined more than 30 class I OR genes from all 4 subgroups, Fig. 3B and data not shown), including tetrapod-specific class I subgroup α, were preferentially expressed in the MC (aquatic olfactory system) whereas the class II ORs were exclusively expressed in the PC as reported by Freitag *et al.* [10] (Fig. 3B). One significant exception was the OR1α5 gene (JGI; e_gw1.2098.6.1) which belongs to the tetrapod-specific subgroup of class I OR, was equally expressed in both the MC and the PC. Our results showed that XtOR2η genes were differentially expressed in the adult olfactory system (Fig. 3B). These genes were preferentially expressed in the MC. No or very low expression was detected in the PC; levels were comparable to levels of expression of each single class I OR (Fig. 3B).

### WISH analysis of OR2η expression in the OSN

**Figure 4:**
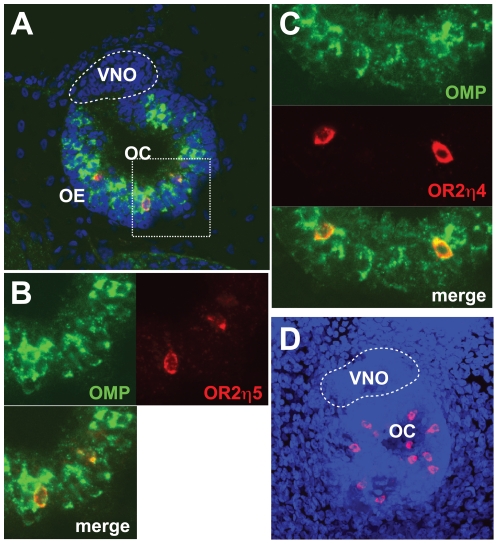
Whole mounts *in situ* hybridization for the XtOR2η4 and XtOR2η5 gene in the olfactory cavity of stage 47 tadpoles. XtOR2η RNA and OMP were detected by Cyanine 3 (red) and fluorescein (green), respectively, and the nucleus was stained with Dapi (blue). A–C: A confocal image of the olfactory cavity. A: XtOR2η5 expression (red). B: Enlargement of dotted square region in panel A. C: XtOR2η4 expression (red). D: A z-stack of all optical planes. XtOR2η4 positive cells (red). OC; olfactory cavity, OE; olfactory epithelium, VNO; veromonasal organ.

If OR2η are involved in odorant detection they should be expressed in the OSN. Thus, we performed WISH of the OC of the tadpole to determine whether OR2 gene expression was limited to the OSN. The small size (1070+/−183 cells (n = 10) in the OE) of the OC of stage 47 tadpoles enabled us to analyze gene expression in the entire organ with a confocal microscope. At this stage there is only one pair of aquatic OC in the tadpole [17]. We chose two OR2η genes for this experiment because of their distinct expression profile in the tadpole. OR2η5 was exclusively expressed in the olfactory organ and OR2η4, which, besides being expressed in the nose, was expressed in other organs such as the brain and the skin. Confocal microscopic analysis clearly demonstrated colocalization of these two OR2η genes and the OMP gene which is regarded as a good molecular marker of mature OSN [28] (Fig. 4A–C). OMP expression was hardly detected in the vomeronasal organ in this stage tadpole (Fig. 4A). Thus, at least two OR2η genes, so far tested, XtOR2η4 and XtOR2η5 were specifically expressed in the OSN in the larval OC. Each OC contained on average 8.9±2.8 (s.d., n = 24) XtOR2η4-positive and 6.0±3.6 (n = 22) XtOR2η5-positive OSN cells (Fig. 4D). The expression was hardly detected in the vomeronasal organ (Fig. 4B). The XtOR2η4- and XtOR2η5-expressing cells were randomly distributed in the OC (data not shown).

## Discussion

The OR2 family was recently identified by *in silico* genome research in the teleost fish and the frog as a close but distinct group of the OR gene super family [14], [16]. Overall sequence homology between OR2 and OR1 (class I and class II ORs), which have 7 transmembrane domains [16], suggests that OR2 is also involved in odorant reception. However, because of the lack of expression data in OSN, the odorant receptor function of OR2 remains unclear. The scope of this paper was to investigate expression patterns of these genes. Our results did not demonstrate direct evidence of the involvement of these receptors in odorant detection. However, we have provided pertinent data to support this hypothesis for OR2η.

### OR2 genes in the X. tropicalis genome

The XtOR2η genes were closely related (39–100% identity in amino acid sequences) and made a gene cluster with one exception. XtOR2η8 was located outside the OR2η cluster. This exceptional XtOR2η8 had a 98% nucleotide identity in the cDNA coding region to that of XtOR2η7 located in the cluster, suggesting that the XtOR2η8 gene duplicated from the XtOR2η7 gene and translocated. OR genes are thought to have increased in number from a small number of ancestor genes by duplication and translocation in the evolution process [32]. Thus, XtOR2η8 is probably one example of the evolution process. We also identified two OR2 genes of two distinct subgroups (κ and θ) outside the OR2η cluster in the genome. However, we found no significant expression of these two genes in the nasal cavities. Thus, OR2κ and θ genes are probably not odorant receptors. The non-OR function of OR2κ and θ was also suggested by Niimura [9] based upon their expression in only non-olfactory tissues [33] and their distinct evolutionally dynamics from the OR genes [9]. In fact, Alioto and Ngai [16] did not identify these highly divergent groups as odorant receptors in the fish genome.

OR2η genes were preferentially expressed in the olfactory system and some other organs (see below) with one exception. Non-specific weak expression of the XtOR2η7 and/or 2η8 gene was detected in all organs tested. Such expression of the OR genes in a broad range of organs has been reported [34], and is thought to be a result of neutral or nearly neutral mechanisms such as small DNA sequence changes in regulatory regions [35], [36]. The OR2η8 gene might have lost its regulatory region by translocation, resulting in the ectopic expression.

### Expression in the olfactory system

Present data showed the co-localization of the expression of two XtOR2η genes so far tested (XtOR2η4 and XtOR2η5) with the OMP gene in the OE in the tadpole. Because the OMP is a marker of mature OSN, this is the first demonstration at the cellular level of the expression of representatives of the XtOR2η gene family by OSN. Because the tadpole is an aquatic animal it is reasonable to suppose that these receptors are involved in aquatic olfaction in the tadpole. We found no particular spatial concentration of the OR2η4- and 5-expressing cells. The distribution of tested OR2η-expressing OSN in the larval OC is probably random. This suggests that OR2η-expression is possibly regulated in a stochastic manner similar to that in other OR genes [37], [38].

In the adult, the MC is known to express class I ORs which detect water-soluble odorants [10], [12]. Our qPCR analysis also showed that most class I OR genes are preferentially expressed in the MC. In this context, preferential expression of OR2η in the MC suggests that like class I ORs they have a water-soluble odorant receptor function similar to the class I ORs. This hypothesis is well supported by the fact that the OR2 family is solely found in aquatic animals such as fish and amphibian frogs. In contrast to this, class II ORs are exclusively expressed in the PC ([10], [18], this paper) and thought to recognize volatile ligands [12]. In the mammalian genome the receptors for water-borne odorants such as OR2η and most class I ORs were selectively lost during tetrapod evolution [14]–[16]. Surprisingly, our data showed that like other class I ORs the mammalian group of class I OR (α and β) was preferentially expressed in the adult MC with one exception: class I ORα5 which is expressed in both the MC and the PC. These results are inconsistent with the hypothesis according to which the mammalian class I ORα and probably β function as receptors for air-borne odorants [9], [14], [15]. One alternative hypothesis could be that during tetrapod evolution, some class I receptors acquired the ability to bind to volatile ligands and subsequently they expanded the number of genes in the genome for adaptation to terrestrial life.

### Expression in other organs

The expression of OR in organs other than olfactory organs is not exceptional and has been reported in other vertebrates. In mammals and birds, OR genes are expressed in the telencephalon [39] and the olfactory bulb [40], [41] during early development. OR proteins are expressed on the axon termini in the olfactory bulb [42], [43], where OR is thought to be involved in axonal guidance of the OSNs to their glomerular targets [44]–[46]. It is also well known that ORs are expressed on the surface of sperm cells and play a role in chemotactic behavior of spermatozoa by the reception of sperm attractant molecules coming from the oviduct [47]–[50]. In this respect it is not surprising that in this study, we found expression of XtOR2η1, 2/3, 4 (and may be 7/8) genes in the brain or all XtOR2η genes except for XtOR2η6 in the testes. As for mammals, such expression in the brain may possibly be involved in development and axonal guidance. The testicular expression could be related to chemotactic behavior of spermatozoa to eggs. If this is true, one of the putative ligands for OR2ηs on the sperm surface could be allurin protein which is related to mammalian sperm binding proteins [51] and recently identified as a sperm chemoattractant in *X. laevis* and *tropocalis* [52], [53].

More puzzling is the expression of XtOR2η4 in the skin. It is known that chemosensory cells, the so-called solitary chemosensory cells, are distributed in the epidermis over the body surface in fish [54]–[56] and in frog tadpoles [57]. OR expression in these cells is not yet clear. However, it may be possible to hypothesize that OR has a chemoreceptor function in these cells. In future studies, it is necessary to identify XtOR2η4-expressing cells in tadpole skin to examine this hypothesis.

Our results demonstrate that OR2η genes in *Xenopus* display different expression patterns. At least two ORηs (XtOR2η4 and 5) are expressed in OSN in the larval olfactory system, suggesting involvement in aquatic olfaction at this stage. In the adult, OR2ηs are preferentially expressed in the MC (qPCR experiments), which responds to water-soluble odorants. Thus, the hypothesis of involvement of OR2η in aquatic olfaction is strong enough to suggest that the physiology should be investigated in future work. Several OR2η genes are expressed in non-olfactory tissues such as the brain and the skin in the larva, besides being expressed in the olfactory organ. In the adult, most OR2η are expressed in the testes and some in the brain. Therefore, these OR2ηs may also have other functions, in addition to olfaction in the nose, such as developmental functions in the brain, chemosensory functions in the skin, and chemotaxis of sperm. In this respect, they share this peculiar feature with mammalian ORs. Further studies of OR2ηs will provide important insights into various OR functions as well as the evolution of chemosensory receptors. Moreover, the study of receptors for water-soluble odorants may important for fishery production.
